# Atezolizumab Induces Necroptosis and Contributes to Hepatotoxicity of Human Hepatocytes

**DOI:** 10.3390/ijms241411694

**Published:** 2023-07-20

**Authors:** Yukinori Endo, Katie L. Winarski, Md Sanaullah Sajib, Anna Ju, Wen Jin Wu

**Affiliations:** Division of Biotechnology Review and Research 1, Office of Biotechnology Products, Office of Pharmaceutical Quality, Center for Drug Evaluation and Research, U.S. Food and Drug Administration (FDA), Silver Spring, MD 20993, USA; yukinori.endo@fda.hhs.gov (Y.E.); katie.winarski@gmail.com (K.L.W.); sajib1989@gmail.com (M.S.S.); annaju@berkeley.edu (A.J.)

**Keywords:** atezolizumab, PD-L1, immune checkpoint, immune checkpoint inhibitors, hepatotoxicity, liver injury, necroptosis, RIP3, necrosome, necrostatin-1

## Abstract

Atezolizumab is an immune checkpoint inhibitor (ICI) targeting PD-L1 for treatment of solid malignancies. Immune checkpoints control the immune tolerance, and the adverse events such as hepatotoxicity induced by ICIs are often considered as an immune-related adverse event (irAE). However, PD-L1 is also highly expressed in normal tissues, e.g., hepatocytes. It is still not clear whether, targeting PD-L1 on hepatocytes, the atezolizumab may cause damage to liver cells contributing to hepatotoxicity. Here, we reveal a novel mechanism by which the atezolizumab induces hepatotoxicity in human hepatocytes. We find that the atezolizumab treatment increases a release of LDH in the cell culture medium of human hepatocytes (human primary hepatocytes and THLE-2 cells), decreases cell viability, and inhibits the THLE-2 and THLE-3 cell growth. We demonstrate that both the atezolizumab and the conditioned medium (T-CM) derived from activated T cells can induce necroptosis of the THLE-2 cells, which is underscored by the fact that the atezolizumab and T-CM enhance the phosphorylation of RIP3 and MLKL proteins. Furthermore, we also show that necrostatin-1, a necrosome inhibitor, decreases the amount of phosphorylated RIP3 induced by the atezolizumab, resulting in a reduced LDH release in the culture media of the THLE-2 cells. This finding is further supported by the data that GSK872 (a RIP3 inhibitor) significantly reduced the atezolizumab-induced LDH release. Taken together, our data indicate that the atezolizumab induces PD-L1-mediated necrosome formation, contributing to hepatotoxicity in PD-L1^+^-human hepatocytes. This study provides the molecular basis of the atezolizumab-induced hepatotoxicity and opens a new avenue for developing a novel therapeutic approach to reducing hepatotoxicity induced by ICIs.

## 1. Introduction

Monoclonal antibodies (mAbs) and their related products have become a new class of therapeutic drugs for cancer treatment [1,2,3,4]. MAbs targeting immune checkpoint molecules or immune checkpoint inhibitors (ICIs) are one of the greatest breakthroughs for antibody-mediated cancer therapy to prolong patient survival in many different solid malignancies [5,6].

The CTLA-4, PD-1, PD-L1, and LAG-3 are well-understood targets of ICIs in the T cell immune system [5,6,7,8]. The programmed death 1 (PD-1) and the cytotoxic T-lymphocyte-associated antigen 4 (CTLA-4) are negative regulators of the T cell immune system [5,7]. The programmed death ligand 1 (PD-L1) and programmed death ligand 2 (PD-L2) are ligands for PD-1, and the CTLA-4 binds B7 [5,7]. The PD-1/PD-L1, PD-1/PD-L2, and CTLA-4/B7 axes inhibit T cell proliferation and activation to shut down the T cell immune system and prevent autoimmunity [5]. On the other hand, many different metastatic tumors upregulate the PD-L1 expression to take an advantage of the negative regulation of the T cell immune system by the immune checkpoint molecules [9,10,11,12,13], and the PD-L1-overexpressed tumors may be associated with poor prognosis [14,15,16]. To date, the FDA has approved several ICIs targeting four checkpoint proteins. These include anti-CTLA-4 (ipilimumab), anti-PD-1 (nivolumab, pembrolizumab, and cemiplimab), anti-PD-L1 (atezolizumab, duravalumab, and avelumab), and anti-LAG-3 (relatlimab) [8,17]. Despite these great successes in cancer therapy, restarting the antitumor activity of the immune system is frequently associated with increased pro-inflammatory cytokines, including IFNγ, IL-1, IL-6, MCP-1, and TNFα in the serum of patients treated with ICIs [6,18,19,20,21,22].

Because checkpoint proteins regulate immune tolerance, the adverse events induced by ICIs are often considered an autoimmune phenomena (called the immune-related adverse event (irAE)) [23,24]. However, the detailed mechanism of irAE is poorly understood. Among the adverse events induced by ICIs, liver injury or hepatotoxicity defined as grade 3 or greater alanine aminotransferase (ALT) elevation is one of dose-limiting factors (DLF). In randomized clinical trials, the incidence of all-grade ALT was 3–9% in the case of anti-CTLA-4 (ipilimumab and tremelimumab), 1.8–7.1% in anti-PD-1 (nivolumab and pembrolizumab), and 0.9–4.0% in anti-PD-L1 (atezolizumab, duravlumab, and avelumab), and all-grade ALT was increased to 17.6–22.3% in a combination of ipilimumab and nivolumab [24]. Furthermore, when combined with chemotherapeutic drugs, the incidence of all-grade ALT (or grade 3–4 ALT) jumped up to 46.4% (10.7% of grade 3–4 ALT) in ipilimumab and 17% (5% of grade 3–4 ALT) in atezolizumab [24]. Interestingly, among ICIs targeting PD-L1, there were differences in the percentage of the incidence of all-grade ALT (or grade 3–4 ALT). The atezolizumab administration showed 5% of all-grade ALT (1% of grade 3–4 ALT), while 1.1% (1.1% of grade 3–4 ALT) in avelumab and 0.2% (0.2% of grade 3–4 ALT) in durvalumab [24,25]. These data suggest that hepatotoxicity occurs dependent on ICI-target molecules and monoclonal antibodies among same ICI-targets. However, the mechanisms of the ICI-induced hepatotoxicity (ICIH) remain to be elucidated.

Adverse events induced by targeted drugs, including monoclonal antibodies and their related products, are categorized into on-target toxicity and off-target toxicity [26]. PD-L1 is categorized as an immune checkpoint. It, however, is expressed on the tumor cell surface and the cell surface of normal tissues, such as hepatocytes. While it has not been reported that targeting PD-L1 expressed on hepatocytes could cause cellular damage and liver injury, direct targeting PD-L1 by anti-PD-L1 antibody (atezolizumab) may contribute to hepatotoxicity and ultimately lead to liver injury.

Necroptosis is a caspase-independent programmed cell death termed the programmed necrosis [27,28]. Morphologically, necrotic cells display some common features, including increasingly translucent cytoplasm, cell plasma membrane rupture, swelling of organelles, and increase in cell volume [28,29]. Necroptosis harbors unique molecular characteristics including receptor interacting protein 1 (RIP1), RIP3, and MLKL [30,31]. Among various stimuli, including TNFα, Fas, TRAIL, IFNα, and IFNβ, the TNFα/TNFR signaling pathway has been studied as a prototype of necroptosis inducer [30]. Upon binding to TNFR on the cell surface, TNFα initiates RIP1 to recruit RIP3 and to form a complex of RIP1/RIP3, where RIP1 becomes phosphorylated by RIP3 followed by the RIP3 auto-phosphorylation [30]. Subsequently, phosphorylated RIP1/RIP3 recruits MLKL and forms a complex termed the necrosome. Then, phosphorylated MLKL translocates from the cytoplasm to the plasma membrane and permeabilizes the plasma membrane, resulting in the swelling of the cell contents and organelles to cause cell death [29,30,32]. However, it remains unclear if necroptosis is involved in the ICI-induced liver injury.

In this study, we revealed a novel mechanism of hepatotoxicity induced by the atezolizumab and a conditioned medium (T-CM) derived from the activated T cells. We found that treatment with either the atezolizumab or the activated T-CM caused hepatotoxicity of human hepatocytes. We provided a number of lines of evidence that demonstrate that necroptosis is a potential mechanism of hepatotoxicity induced by the atezolizumab or T-CM, and that necrostatin-1 may be a useful drug for combination therapy with the atezolizumab or other ICIs to reduce the liver injury.

## 2. Results

### 2.1. PD-L1 Is Expressed in Human Hepatocytes

**Figure 1:**
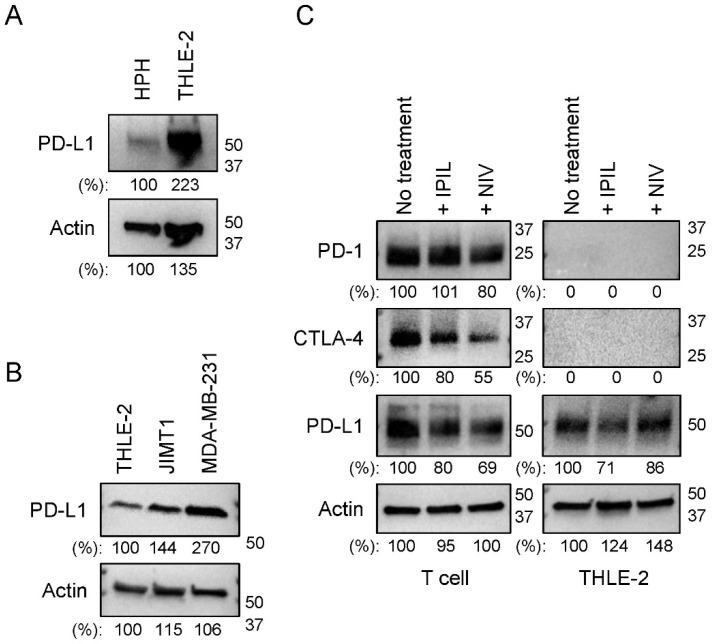
Human hepatocytes express PD-L1 but not PD-1 and CTLA-4. (**A**) The levels of PD-L1 expression were evaluated by Western blotting in whole cell lysates (WCLs) of human primary hepatocytes (HPH) and human hepatocytes (THLE-2 cells). (**B**) The levels of PD-L1 were evaluated by Western blotting in WCLs of THLE-2, JIMT1, and MDA-MB-231 cells. (**C**) The levels of PD-L1, PD-1, and CTLA-4 expression were evaluated by Western blotting in WCLs of activated T cells and THLE-2 cells after 24 h after treatment of 100 µg/mL ipilimumab (IPIL), nivolumab (NIV), and left untreated. Western blotting panels shown in this figure are representative of at least two or three independent experiments.

To investigate the possibility that the atezolizumab, an anti-PD-L1 monoclonal antibody, induces hepatoxicity, the PD-L1 expression in the THLE-2 cells and human primary hepatocytes (HPH) was examined using a Western blot analysis. The expression level of PD-L1 in HPH was lower than that in the THLE-2 cells (Figure 1A), consistent with previous observations that the PD-L1 expression is kept low in the liver primary hepatocytes [33,34]. We further compared the expression of PD-L1 in the THLE-2 cells with two breast cancer cell lines, MDA-MB-231 and JIMT1. As shown in Figure 1B, the level of PD-L1 expression in MDA-MB-231 was higher than that in the THLE-2 cells. The PD-L1 expression in human hepatocytes was also examined using fluorescent immunostaining. Supplemental Figure S1 shows the detection of the PD-L1 expression in the human hepatocyte cell line (THLE-2). Next, we examined the expression of two additional immune checkpoint regulators, PD-1 and CTLA-4, in human hepatocytes. As shown in Figure 1C, the PD-1 and CTLA-4 expressions were detected in the activated T lymphocytes but were not detected in the THLE-2 cells (Figure 1C). It is noted that ipilimumab (IPIL) and nivolumab (NIV) decreased the expression levels of CTLA-4 and PD-L1 in the activated T cells treated with IPIL and NIV for 24 h. Taken together, human hepatocytes express PD-L1 and are likely targeted by the atezolizumab, although its expression level of PD-L1 is relatively low compared with that in triple-negative breast cancer (TNBC) cells, MDA-MB-231.

### 2.2. Atezolizumab Is Capable of Inducing Cytotoxicity in Human Hepatocytes

**Figure 2:**
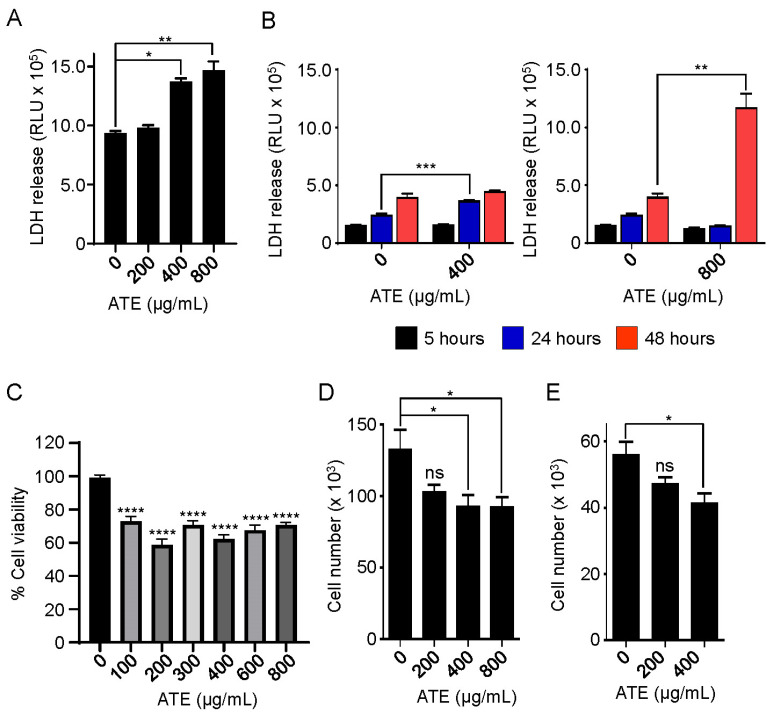
Atezolizumab alone causes cytotoxicity in human hepatocytes. (**A**) The levels of LDH released in the cell culture medium of human primary hepatocytes (HPH) were measured after the cells were treated with indicated concentration of ATE for 24 h. (**B**) The levels of LDH released in the cell culture medium of THLE-2 were measured after the cells were treated with indicated concentrations of ATE for 5, 24, and 48 h. (**C**) Cell viability was examined using MTT assay in THLE-2 cells after the cells were treated with indicated concentrations of ATE for 48 h. (**D**) Cell growth was examined in THLE-2 cells after the cells were treated with indicated concentrations of ATE for 48 h. (**E**) Cell growth was examined in THLE-3 cells after the cells were treated with indicated concentrations of ATE for 48 h. Hepatocyte cytotoxicity assays shown in this figure are representative of at least two or three independent experiments. * *p*-value < 0.05, ** *p*-value < 0.01, *** *p*-value < 0.001, **** *p*-value < 0.0001, and ns indicates no significant change.

The atezolizumab is directed against PD-L1 expressed on solid tumors including advanced/metastatic triple-negative breast cancer, resulting in disengagement of the interaction of PD-L1 and PD-1 to reactivate the T cell immune system [5,35]. In our recent study [36], we showed that PD-L1 plays an important role in regulating FAK phosphorylation involved in the cell migration and proliferation of MDA-MB-231 and BT-20 breast cancer cells [36]. Additionally, Saleh et al. (2019) showed that the atezolizumab downregulates the signaling pathways associated with tumor growth, metastasis, and hypoxia in MDA-MB-231 cells [37]. Thus, in this study, we hypothesized that the atezolizumab alone was able to induce cytotoxicity in human hepatocytes. We first examined if atezolizumab treatment induced the release of alanine aminotransferase (ALT) in the cell culture conditioned medium as an indicator for cytotoxicity in MDA-MB-231 cells. ALT is also called glutamic pyruvate transaminase. *GTP* genes, including *GTP1* and *GTP2*, are overexpressed in a variety of cancer cell lines, including breast cancer cells (The Human Protein Atlas: www.proteinatlas.org, accessed on 8 July 2023). The GTP2 protein is also involved in the tumorigenesis of breast cancer cells [38]. MDA-MB-231 cells were treated with either 200 µg/mL atezolizumab (ATE) or ipilimumab (IPIL), nontreated or treated with 0.5 µM doxorubicin as a positive control, and then the ALT activity in the conditioned medium was examined at 5 and 24 h time points. As shown in Supplemental Figure S2, the ALT activity was increased in the conditioned medium from cells treated with both the doxorubicin and atezolizumab at the 24 h time point, while ipilimumab failed to induce the ALT release in the cell-culture conditioned medium. These results suggest a possibility that the atezolizumab is able to induce injury of PD-L1-positive hepatocytes. We next examined if the atezolizumab induced ALT release in the conditioned medium in human hepatocytes (THLE-2). ALT, however, was not detected in the THLE-2 cells. This was not an unexpected result, as ALT is hardly detected in human hepatocytes in vitro. Therefore, we decided to examine lactate dehydrogenase (LDH) leaked in the conditioned medium from human primary hepatocytes (HPH). As shown in Figure 2A, LDH was detected in the conditioned medium of the HPH in a dose-dependent manner after treated by the atezolizumab for 24 h. From the results, a relatively high concentration of the atezolizumab such as 400 or 800 µg/mL was required for the atezolizumab to induce cellular injury of human primary hepatocytes, while 200 µg/mL of atezolizumab failed to do so. The higher concentrations of the atezolizumab to induce the cytotoxicity in this study are consistent with a range of the maximum atezolizumab serum concentration (414 to 891 µg/mL) in steady state in patients treated with a dose of 1200 mg atezolizumab every three weeks (q3w) [39]. We also found that LDH was detected in the conditioned medium of the THLE-2 cells treated with 400 µg/mL atezolizumab for 24 h and 800 µg/mL atezolizumab for 48 h treatment (Figure 2B). Based on our data, the trends of LDH release in the culture media suggest that the atezolizumab likely induces cellular injury of hepatocytes. Supporting the detection of LDH released in the cell culture medium after atezolizumab treatment, the viability of hepatocytes was reduced when the THLE-2 cells were treated by the atezolizumab at all concentrations indicated for 48 h (Figure 2C). Consistent with the data shown in Figure 2A–C, the growth of the THLE-2 and THLE-3 cells was inhibited when these cells were treated with different doses of the atezolizumab for 48 h in a dose-dependent manner (Figure 2D,E, respectively). Taken together, these results suggest that the atezolizumab is capable of inducing cytotoxicity, thus causing hepatotoxicity in human hepatocytes.

### 2.3. Necrosome Is Formed after Treatment of Atezolizumab in THLE-2 Cells

**Figure 3:**
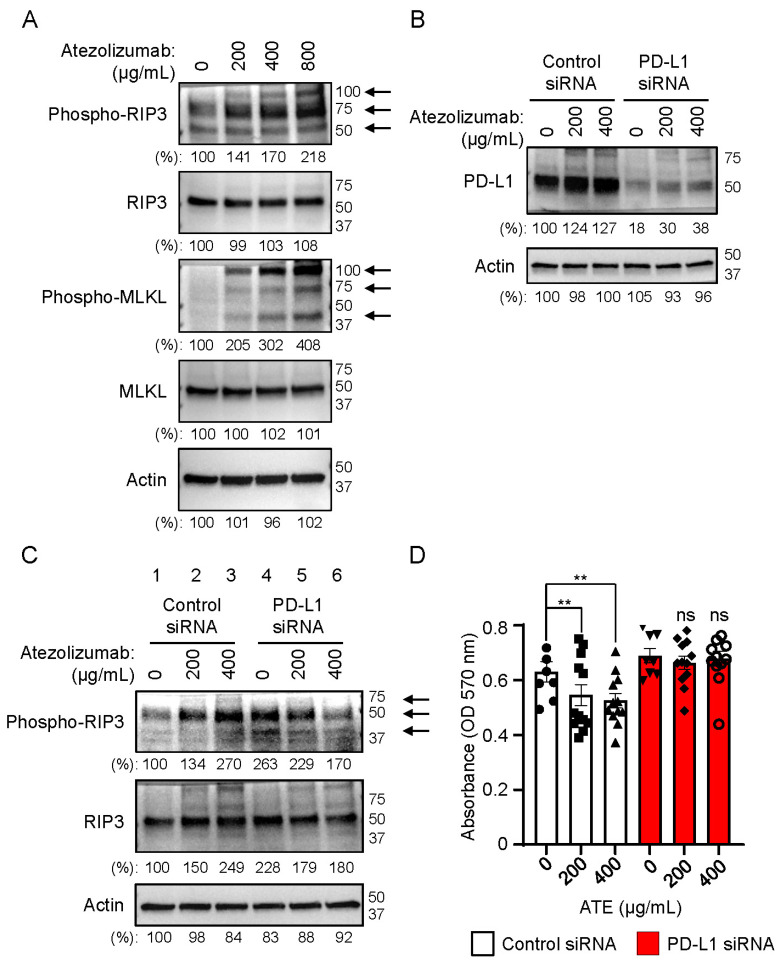
Phosphorylated RIP3 and MLKL are increased in atezolizumab-treated human hepatocytes (THLE-2 cells) in a dose-dependent manner. (**A**) The levels of phosphorylated RIP3, RIP3, phosphorylated MLKL, and MLKL expression were evaluated by Western blotting in WCLs of THLE-2 cells after the cells were treated with either indicated concentrations of ATE or left untreated as a control. Arrows indicate oligomerized phosphorylated RIP3 and MLKL, respectively. (**B**) Knockdown efficiency of PD-L1 expression was examined by Western blotting in WCLs of THLE-2 cells 48 h after transfection of control siRNA and PD-L1 siRNA. Both control siRNA and PD-L1 siRNA-transfected cells were treated with indicated concentrations of atezolizumab for 24 h. (**C**) The levels of phosphorylated RIP3 and RIP3 expressions were evaluated by Western blotting in the WCLs of THLE-2 cells used in (**B**). Arrows indicate oligomerized phosphorylated RIP3. (**D**) Cell viability was examined in THLE-2 cells after the control and PD-L1 siRNA-transfected cells were treated with indicated concentrations of atezolizumab for 24 h. Western blotting panels shown in this figure are representative of at least two or three independent experiments. Black rounds, cubes, triangles, inverted triangles, rhombuses, hollow circles indicate individual sample tested. ** *p*-value < 0.01, and ns means no significant change.

To explore the mechanism by which the atezolizumab induced hepatotoxicity in human hepatocytes, we found that cleaved caspase 3 was not observed after treatment with the atezolizumab in the THLE-2 cells (Supplemental Figure S3). These data suggested that apoptosis might not be involved in the process of hepatotoxicity induced by the atezolizumab in human hepatocytes. To search for a different mechanism of hepatotoxicity induced by the atezolizumab, we investigated if necroptosis, a different cell death pathway, was involved in the hepatotoxicity process induced by the atezolizumab in human hepatocytes. RIP3 is an indispensable molecule for necroptosis and is one of the components of the necrosome [30]. The RIP1 activation leads to RIP3 homo-oligomerization and auto-phosphorylation [29,40]. We found that phosphorylated RIP3 proteins, which were shown as multiple protein bands with different molecular weight, were increased in a dose-dependent manner in the THLE-2 cells treated with different doses of the atezolizumab for 48 h (Figure 3A). In addition to RIP3, MLKL is another important necrosome component [31]. MLKL is oligomerized upon its phosphorylation by RIP3 [29,32,41]. As shown in Figure 3A, phosphorylated MLKL proteins, which were also shown as multiple protein bands with different molecular weight, were also increased in the THLE-2 cells treated with the atezolizumab in a dose-dependent manner for 48 h. These results strongly suggested that necrosome was involved in hepatotoxicity induced by the atezolizumab in human hepatocytes. Next, we addressed the question whether necrosome-mediated cytotoxicity occurred via PD-L1. The PD-L1 gene expression in the THLE-2 cells was knocked down using a siRNA technology. The data displayed in Figure 3B confirmed that the knockdown efficiency of PD-L1 was 80%, and the cells were then treated with different concentrations of the atezolizumab for 24 h. As shown in Figure 3B, the PD-L1 expression was slightly increased in a dose-dependent manner when the cells were treated with different concentrations of the atezolizumab both in the control siRNA and the PD-L1 siRNA-treated cells (Figure 3B). We next examined the levels of phosphorylated RIP3 in the PD-L1-knocked-down THLE-2 cells. Figure 3C shows that the phosphorylation of RIP3 was increased in the control siRNA-treated cells when the cells were treated with atezolizumab in a dose-dependent manner for 24 h. However, the level of phosphorylated RIP3 in the PD-L1-knocked-down cells was unexpectedly increased in the PD-L1-knocked-down cells that were not treated with the atezolizumab (Figure 3C, lane 4 compared with lane 1). These results suggest that PD-L1 likely plays a role in preventing the activation of RIP3, which triggers cell death via necroptosis in human hepatocytes. This hypothesis was underscored by the fact that the levels of phosphorylated RIP3 were not further increased in the PD-L1-knocked-down cells treated with the atezolizumab (Figure 3C, lanes 4, 5, and 6). These results indicate that PD-L1 plays a critical role in cells to prevent the RIP3 activation. Upon binding to the atezolizumab, PD-L1 becomes dysfunctional, resulting in increased phosphorylation of RIP3 in human hepatocytes. This observation was further supported by the knockdown of PD-L1, which promoted RIP3 phosphorylation. These results suggest that the hepatotoxicity induced by the atezolizumab is mediated via PD-L1. We next addressed the question whether the decreased cell viability caused by the atezolizumab could be rescued by PD-L1 knocked-down in human hepatocytes. As shown in Figure 3D, the decreased cell viability caused by the atezolizumab observed in the control THLE-2 cells was rescued in the PD-L1-knocked-down cells treated with 400 µg/mL atezolizumab for 24 h (Figure 3D). These results support the idea that hepatotoxicity induced by the atezolizumab is mediated by PD-L1 in human hepatocytes.

### 2.4. PD-L1 Expression Is Upregulated by Cytokines Secreted from Activated T Lymphocytes

**Figure 4:**
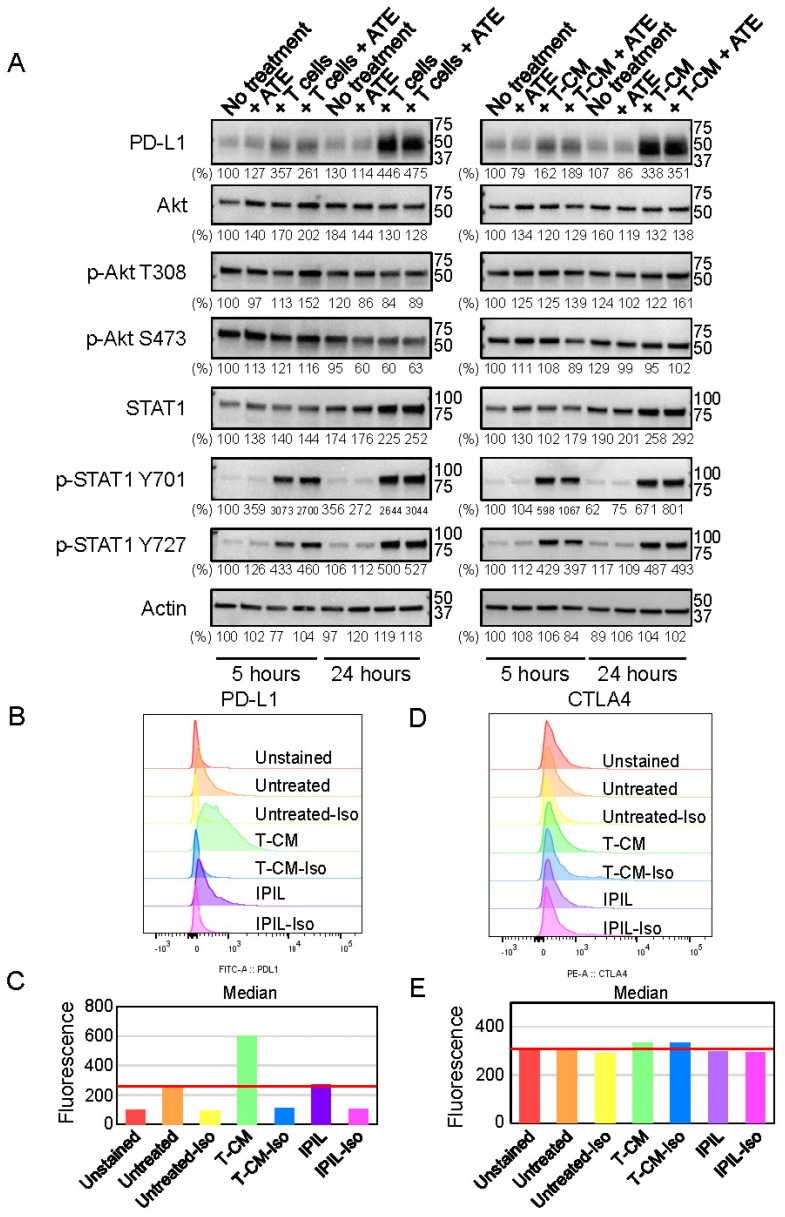
T-cell-derived conditioned medium (T−CM) enhances expression level of PD−L1 but not CTLA−4 in human hepatocytes (THLE−2 cells). (**A**) The levels of PD−L1, phosphorylated Akt (T308 and S473), total Akt, phosphorylated STAT1 (Y701 and Y727), and total STAT1 expression were evaluated by Western blotting in WCLs of THLE-2 cells after the cells were co-cultured with activated T cells (1:3 ratio of THLE−2 cells: activated T cells) or cultured in the presence of conditioned medium (T−CM) (3:1 ratio of THLE-2 cell culture medium: T−CM) derived from activated T cells for 5 h and 24 h. (**B**) The levels of PD−L1 expression were evaluated by flow cytometry in THLE−2 cells after the cells were treated with T−CM (3:1 ratio), 100 µg/mL ipilimumab (IPIL), and untreated control. Red: unstained; orange: untreated control; yellow: untreated-Isotype IgG control; green: T−CM; blue: T−CM-Isotype IgG control; purple: IPIL; pink: IPIL-Isotype IgG control. (**C**) Quantitative analysis of flow cytometry data shown in (**B**). (**D**) The levels of CTLA−4 expression were evaluated by flow cytometry in THLE-2 cells after the cells were treated with T-CM (3:1 ratio), 100 µg/mL ipilimumab (IPIL), and untreated control. Red: unstained; orange: untreated control; yellow: untreated-Isotype IgG control; green: T−CM; blue: T−CM-Isotype IgG control; purple: IPIL; pink: IPIL−Isotype IgG control. (**E**) Quantitative analysis of flow cytometry data shown in (**D**). Western blotting panels and flow cytometry analysis shown in this figure are representative of at least two or three independent experiments.

It has been shown that the PD-L1 expression in cancers and hepatocytes was increased by cytokines such as IFNs and TNFs in the tumor microenvironment and in vitro, respectively [33,42]. Since immune checkpoint inhibitors such as the atezolizumab, ipilimumab, and nivolumab maintain the activated state of T lymphocytes or reactivate them, these cytokines such as IFNs and TNFs are abundantly secreted by the activated T cells [6,18,19,20,21,22]. Therefore, we examined if secreted cytokines from the activated T cells upregulated the PD-L1 expression in human hepatocytes. The THLE-2 cells were co-cultured with preactivated T cells, and then the levels of PD-L1, total Akt, p-Akt (T308 and S473), total STAT1, and p-STAT1 (Y701 and Y727) were evaluated by a Western blot analysis after 5 and 24 h because the PD-L1 expression is regulated by the STAT1-mediated pathway [43]. As shown in the left panel of Figure 4A, p-STAT1 Y701 and Y727 were increased in the THLE-2 cells after being co-cultured with the activated T cells for 5 h. After 24 h incubation, in addition to the further enhanced levels of p-STAT1 Y701 and Y727, the levels of the PD-L1 expression were dramatically increased in the THLE-2 cells when the cells were co-cultured with T cells. The levels of p-Akt (T308 and S473) were not changed (Figure 4A). These results suggested that the STAT1, but not Akt, signaling pathway is involved in upregulating the PD-L1 expression in the THLE-2 cells upon interacting with the activated T cells [43]. To investigate whether the direct cell-to-cell contact between the hepatocytes and activated T cells is involved in upregulating the PD-L1 expression in the THLE-2 cells, the THLE-2 cells were then cultured in the presence of a conditioned medium (T-CM) derived from the activated T cells for 5 and 24 h. As shown in the right panel of Figure 4A, similar patterns were obtained to those observed in the THLE-2 cells when the cells were co-cultured with the activated T cells. These results suggest that cytokines secreted from the activated T cell are involved in the process of increased the PD-L1 expression in the THLE-2 cells. The increased levels of the PD-L1 expression were further examined by flow cytometry. As shown in Figure 4B,C, the level of the PD-L1 expression was increased about threefold compared to that of untreated control cells, whereas the level of the CTLA-4 expression was not changed in the THLE-2 cells even in the presence of T-CM (Figure 4D,E). These results suggest that cytokines secreted from the activated T cells are sufficient to upregulate the PD-L1 expression via the STAT1 signaling pathway in liver tissues.

### 2.5. Conditioned Medium (T-CM) Derived from Activated T Cells Activates RIP3 in Human-Hepatocyte THLE-2 Cells

**Figure 5:**
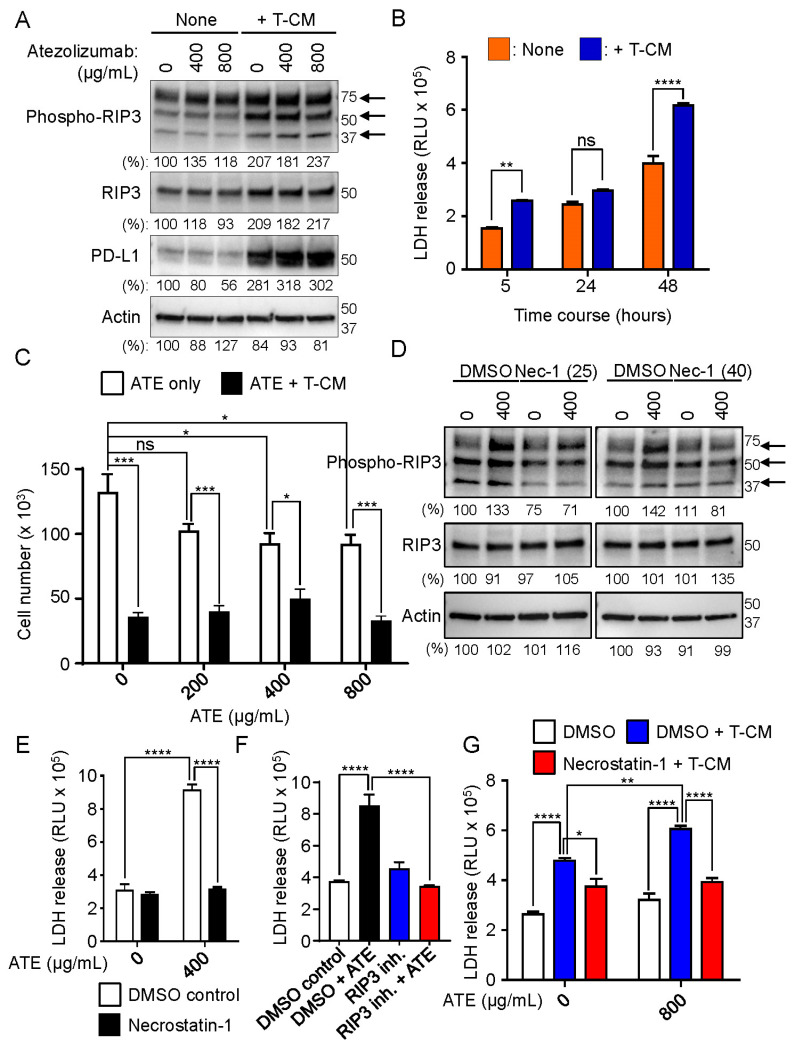
Both atezolizumab and T-CM induce necrosome formation in human hepatocytes (THLE-2 cells), and in turn, the induced necrosome causes hepatotoxicity in THLE-2 cells. (**A**) The levels of phosphorylated RIP3, RIP3, and PD-L1 expression were evaluated by Western blotting in WCLs of THLE-2 cells after the cells were treated with indicated concentrations of ATE for 48 h. Arrows indicate oligomerized phosphorylated RIP3. (**B**) The levels of LDH released in the cell culture medium of human hepatocytes (THLE-2 cells) were measured after treatment of the cells with T-CM (3:1 ratio of THLE-2 cell culture medium: T-CM) at 5, 24, and 48 h time points. (**C**) Cell growth was examined in THLE-2 cells after the cells were cultured in the presence of T-CM (3:1 ratio) in a combination of indicated concentrations of ATE for 48 h. (**D**) The levels of phospho-RIP3 and RIP3 expression was evaluated by Western blotting in WCLs of THLE-2 cells when the cells were treated with 400 µg/mL atezolizumab in the presence/absence of 25 and 40 µM necrostatin-1. Arrows indicate oligomerized phosphorylated RIP3. (**E**) The levels of LDH released in the cell culture medium of human hepatocytes (THLE-2 cells) were measured after the cells were treated with 25 µM necrostatin-1 in the presence or absence of 400 µg/mL atezolizumab for 24 h. (**F**) The levels of LDH released in the cell culture medium of human hepatocytes (THLE-2 cells) were measured after the cells were treated with 3 µM RIP3 inhibitor, GSK872, in the presence or absence of 800 µg/mL atezolizumab for 24 h. (**G**) The levels of LDH released in the cell culture medium of human hepatocytes (THLE-2 cells) were measured after the cells were treated with 50 µM necrostatin-1 in the presence or absence of T-CM (3:1 ratio) for 24 h. Western blotting panels and hepatocyte cytotoxicity assays shown in this figure are representative of at least two or three independent experiments. * *p*-value < 0.05, ** *p*-value < 0.01, *** *p*-value < 0.001, **** *p*-value < 0.0001, and ns indicates no significant change.

Immune checkpoint inhibitors function to reactivate the immune system to attack cancer cells [5,6]. We also found that cytokines secreted from the activated T cells increased the PD-L1 expression in human hepatocytes (THLE-2 cells) (Figure 4). We confirmed that IFNγ and TNFα were included in the T-CM, using a human cytokine array (Supplemental Figure S4). The expressions of the receptors for IFNγ and TNFα, i.e., IFNGR2 and TNFRSF1A/B, respectively, in the human hepatocytes of liver tissues were previously examined by immunohistochemistry (IHC) (The Human Protein Atlas: www.proteinatlas.org, accessed on 8 July 2023). TNFα increases necrosome formation to cause necroptosis by activating RIP3 followed by the RIP3 and MLKL activation upon binding to TNFR1 [30]. We therefore examined whether T-CM activated the RIP3 in the THLE-2 cells. The THLE-2 cells were cultured in the presence or absence of T-CM with a combination of different concentrations of the atezolizumab for 48 h, and then the levels of phosphorylated RIP3, total RIP3, and PD-L1 expression were evaluated by a Western blot analysis. As shown in Figure 5A, phosphorylated RIP3 was increased in the THLE-2 cells treated with the atezolizumab in a dose-dependent manner in the absence of T-CM. In the presence of T-CM, the PD-L1 expression was dramatically increased, and T-CM alone enhanced the level of phosphorylated RIP3 in the THLE-2 cells (Figure 5A). Th atezolizumab did not further increase the levels of phosphorylated RIP3 in the presence of T-CM (Figure 5A). Next, we examined whether T-CM induced cytotoxicity of the THLE-2 cells using an LDH release assay in the cell culture medium of the THLE-2 cells after 5, 24, and 48 h. As shown in Figure 5B, LDH was detected in the cell culture medium at all three time points. These results suggest that T-CM is also able to induce hepatotoxicity in the THLE-2 cells. Furthermore, as a result of the LDH release, the growth of the THLE-2 cells was inhibited in the presence of T-CM. However, it appeared that the atezolizumab did not exhibit a synergistic effect on the inhibition of cell growth induced by T-CM (Figure 5C).

### 2.6. RIP3 Activation Followed by Necrosome Formation Is Involved in Hepatotoxicity Induced by Atezolizumab and T-CM in Human Hepatocytes (THLE-2 Cells)

Finally, we addressed the question if necrosome formation was involved in the hepatotoxicity induced by the atezolizumab. RIP1 is one of the upstream molecules of necrosome formation and activates RIP3 using its Ser/Thr kinase activity [30]. Necrostatin-1 is a potent RIP1 kinase inhibitor [44,45,46]. The levels of phosphorylated RIP3 were examined in the presence of necrostatin-1 in the THLE-2 cells when the cells were treated with either 400 µg/mL atezolizumab or left untreated. As shown in Figure 5D, the atezolizumab increased phosphorylated RIP3 from 1.3 to 1.4 times in the THLE-2 cells. The increased phosphorylated RIP3 induced by the atezolizumab was decreased by necrostain-1 in a dose-dependent manner (Figure 5D). Furthermore, we confirmed that the T-CM-mediated phosphorylation of RIP3 was inhibited by 25 µM necrostain-1 in the THLE-2 cells after 24 h treatment (Supplemental Figure S5). These results indicate that necrostatin-1 blocks the RIP3 activation by blocking the RIP1 kinase activity, followed by inhibiting necrosome formation in the THLE-2 cells. We next examined the effects of necrostatin-1 on hepatotoxicity in the THLE-2 cells using an LDH release assay. As shown in Figure 5E,F, the increased LDH release in the THLE-2 cell culture medium was significantly reduced to the level of control cells treated with 0 µg/mL atezolizumab when the cells were treated with the atezolizumab for 24 h. These results suggest that the RIP3 activation followed by necrosome formation plays a critical role in hepatotoxicity induced by the atezolizumab in the THLE-2 cells. Similar to the results shown in Figure 5E,F, the T-CM-induced increase of LDH release in the THLE-2 cell culture medium was significantly reduced by necrostatin-1 (Figure 5G). These data also support the notion that necrosome formation is responsible for hepatotoxicity induced by cytokines such as TNFα secreted by the activated T cells. Taken together, our data suggest that atezolizumab treatment, as well as cytokines secreted from the activated T lymphocytes can induce the RIP3 activation followed by necrosome formation, resulting in hepatotoxicity in human hepatocytes.

## 3. Discussion

Immune checkpoint inhibitors (ICIs) are developed for the purpose of reactivating the immune system to attack tumor cells by cytokines secreted by the activated T cells [6]. Hepatotoxicity is one of the major causes of dose-limiting toxicity (DLT) for ICI therapeutics [24,25,47]. Therefore, improving the therapeutic index (TI) of next-generation ICIs will not be accomplished unless we obtain a greater understanding of the underlying mechanism of hepatotoxicity.

**Figure 6:**
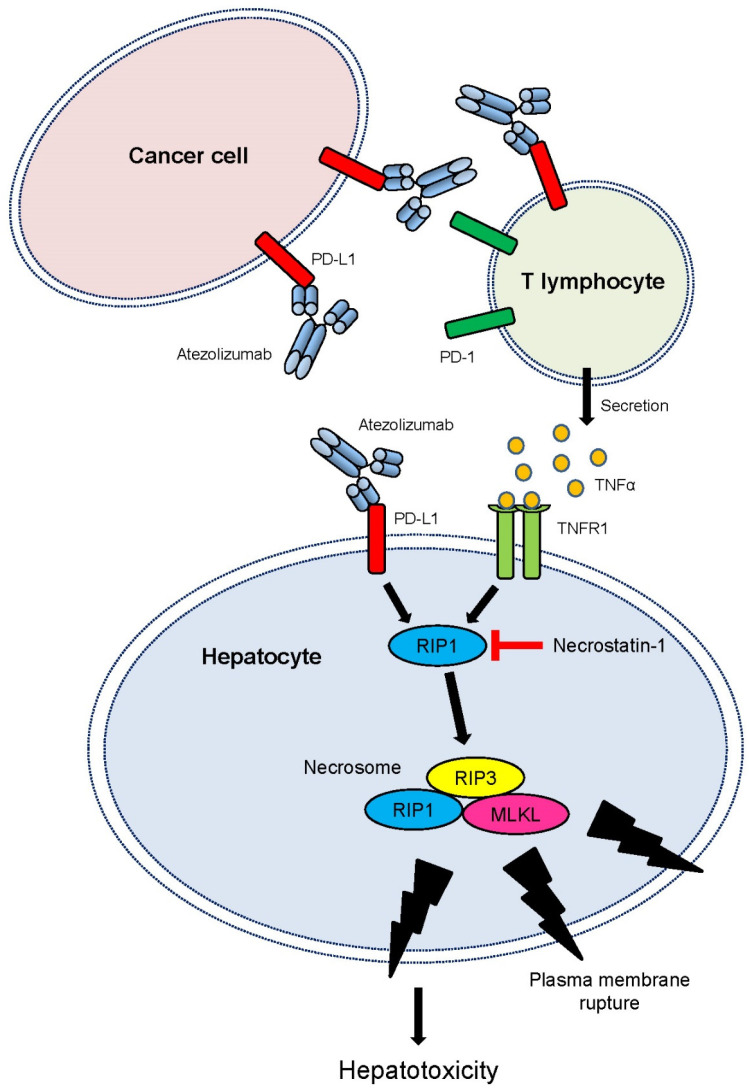
A working model: atezolizumab-induced cytotoxicity. Upon specific binding to PD-L1 on cell surface of hepatocytes, atezolizumab begins to induce RIP1 activation followed by necrosome formation consisting of RIP1, RIP3, and MLKL. Subsequently, the formed necrosome damages cell plasma membrane to cause membrane rupture, resulting in decreased cell viability, cell growth inhibition, and hepatotoxicity. While atezolizumab-mediated direct interaction with PD-L1 in hepatocytes plays a major role in atezolizumab-induced killing of PD-L1-positive hepatocytes, cytotoxicity mediated by activated T cells is also important for necrosome formation and atezolizumab-induced cytotoxicity of liver cells where TNFR1 receives TNFα secreted from activated T cells and induces necrosome formation, resulting in decreased cell viability, cell growth inhibition, and hepatotoxicity by damaging the plasma membrane.

Our study reveals a novel mechanism by which the atezolizumab mediates PD-L1-dependent necroptosis, leading to cytotoxicity of hepatocytes. A working model for the atezolizumab-induced necroptosis and hepatotoxicity, based on several lines of experimental evidence, is presented in Figure 6. Upon binding to PD-L1 expressed on hepatocytes, the atezolizumab induces the RIP1 activation, leading to the formation of RIP1/RIP3 complex, RIP3 autophosphorylation, and necrosome formation including RIP1/RIP3/MLKL. Subsequently, the formation of necrosome leads to decreased cell viability, cell growth inhibition, and hepatotoxicity in human hepatocytes potentially via phosphorylated and oligomerized MLKL that translocates to the plasma membrane, resulting in the plasma membrane rupture. Furthermore, blocking the PD-L1–PD-1 interactions, the atezolizumab triggers the activation of T cells to promote the secretion of a various cytokines including TNFα. This, in turn, further induces necrosome formation, resulting in the plasma membrane rupture and hepatotoxicity (Figure 6).

The involvement of necroptosis in mediating hepatotoxicity may change our current understanding of ICI-induced hepatotoxicity (ICIH), which is believed to be largely considered as an autoimmune-related adverse event [23]. However, it has been shown that there are significant histopathological differences between the ICIH and idiopathic autoimmune hepatitis (iAIH) [24,47,48]. Furthermore, based on the investigations of biopsied livers from patients who received ICIs, e.g., anti-CTLA-4 mAb in monotherapy or in combination with anti-PD-1 mAbs, or anti-PD-1 mAbs in monotherapy, there were also histopathological differences between anti-CLTA-4 and anti-PD-1 mAbs [48]. However, the molecular mechanism underlying ICIH remains to be elucidated. In this study, we propose two potential pathways to cause the ICIH (Figure 6). Each may contribute to inducing the necrosome either via directly binding to PD-L1 expressed on hepatocytes or activating T cells, resulting in hepatotoxicity (Figure 6). This study discovered a new role of RIP3 in its involvement in the ICI-induced hepatotoxicity and liver injury. Our finding is consistent with the previous report that RIP3 knockout mice were protected from alcohol-induced liver injury and secretions of pro-inflammatory cytokines including TNFα [49,50]. These studies suggest that necroptosis via RIP3 might be widely involved in livery injuries induced by alcohol consumption and chemotherapeutic drugs. Additionally, these studies also suggest that damage-associated molecular patterns (DAMPs) leaked from the necroptotic cells has a potential to enhance pro-inflammatory cytokine secretions including TNFα from the immune systems [19,51]. The sources of TNFα in the serum are not only activated T lymphocytes, but also activated monocytes stimulated by DAMPs [18]. Therefore, there is a positive feedback loop to enhance the levels of pro-inflammatory cytokines in the serum [18,20], which may further enhance ICIH.

Necrosome inhibitor necrostatin-1 (Nec-1) specifically targets RIP1 to block necrosome formation induced by ICIs [44,45,46] while the antitumorigenic activity by ICIs should be sustained. This study provides a potential novel therapeutic approach to reduce ICIH by a combination of necrostatin-1 with ICIs, including the atezolizumab, to reduce hepatotoxicity induced by ICIs.

## 4. Materials and Methods

### 4.1. Human Hepatocytes, Breast Cancer Cells, and Therapeutic Drugs

Human primary hepatocytes (HPH) were obtained from ScienCell (cat# 5200, Carlsbad, CA, USA) and maintained in hepatocyte medium (ScienCell, cat# 5201) containing 5% FBS according to the manufacturer’s instruction. Human hepatocytes (THLE-2 and THLE-3) were purchased from ATCC (cat# CRL-2706 and cat# CRL-11233, Manassas, VA, USA, respectively) and were cultured in BEGM SingleQuots medium (Lonza, cat# CC3170, Basel, Switzerland) containing 10% FBS. Breast cancer cell line JIMT1 cells were purchased from DSMZ (Braunschweig, Germany, cat# ACC 589) and cultured in Dulbecco’s modified Eagle’s medium (DMEM) containing 10% FBS. Another breast cancer cell line MDA-MB-231 cells were purchased from ATCC (cat# HTB-26) and cultured in RPMI-1640 containing 10% FBS. Atezolizumab, ipilimumab, nivolumab, and doxorubicin were purchased from the pharmacy at NIH (Bethesda, MD, USA). Necrostain-1 was purchased from MedChemExpress (cat# HY-15760, South Brunswick Township, NJ, USA). RIP3 inhibitor, GSK872, was purchased from Sigma-Aldrich (cat# 530389, St. Louis, MO, USA).

### 4.2. Activated T Lymphocytes Preparation

Human Pan T cells obtained from STEMCELL Technologies (Burnaby, BC, Canada) were cultured and activated according to manufacturer’s instructions. Briefly, the Pan T cells were recovered and cultured on 12-well plates containing 1 mL of RPMI-1640 with 10% FBS, antibiotics, sodium pyruvate, and MEM nonessential amino acids. Pan T cells (1–5 × 10^6^) were then activated with 25 µL of ImmunoCult Human CD3/CD28/CD2 T Cell Activator (STEMCELL technologies, cat# 10970) and 2 µL of human recombinant IL-2 (STEMCELL technologies, cat# 78036) for 3–4 days before proceeding with experiments. In addition, Pan T cells were also isolated from human buffy coat blood or leukopak of healthy donors, which were obtained from NIH (Bethesda, MD, USA). PBMCs were first isolated from the buffy coat blood or leukopak using Ficoll-Hypaque density gradient centrifugation. ACK lysis buffer was used to remove red blood cells from PBMCs. Subsequently, Pan T cells were isolated using EasySep Human Pan T cell isolation kit (STEMCELL Technologies, cat# 17961) according to manufacturer’s instructions. Pan T cells (1–5 × 10^6^) were then activated using 25 µL of the ImmunoCult Human CD3/CD28/CD2 T Cell Activator and 2 µL of the recombinant IL-2 on 12-well plates for 3–4 days before proceeding with experiments.

For co-culture with THLE-2 cells, 6 × 10^5^ activated T cells were co-cultured with 2 × 10^5^ THLE-2 cells (3:1 ratio) on 12-well plates for 5 and 24 h.

The conditioned medium was separately collected from activated T cell culture after removing cells and cell debris by centrifugation and 0.2 µm filtration, and then it is called T-cell-derived conditioned medium (T-CM) in this study. For co-culture with THLE-2 cells, 2 × 10^5^ THLE-2 cells were cultured in T-CM-containing THLE-2 cell culture medium (3:1 ratio of THLE-2 cell culture medium: T-CM) for 5 and 24 h.

Cytokines in T-cell-derived conditioned medium (T-CM) were examined using human XL cytokine array (R&D Systems, cat# ARY022B, Minneapolis, MN, USA).

### 4.3. Western Blotting

The detailed procedures were previously described [52]. Briefly, 5 × 10^5^ THLE-2 cells were seeded on 6-well plates three days prior to cell treatment, after three days incubation the cells were treated with a series of drugs as described in the manuscript. Twenty-four hours after the drug treatment, the cells were washed with PBS and then lysed with an NP40 lysis buffer on ice for 30 min. After centrifugation, the whole cell lysates (WCLs) were subjected to a Western blot analysis under nonreduced condition. Western blotting panels shown in the figures of this manuscript are representative of at least two or three independent experiments. Each cell treatment was coming from a single well. The following primary antibodies were used for a Western blotting analysis: actin (Sigma-Aldrich, cat# A1978), Akt (Cell Signaling Technology, cat# 4691S, Danvers, MA, USA), phospho-Akt (T308) (Cell Signaling Technology, cat# 9275S), phospho-Akt (S473) (Cell Signaling Technology, cat# 4060S), Bcl-2 (Cell Signaling Technology, cat#2870), Beclin-1 (Cell Signaling Technology, cat#3738), cleaved caspase 3 (Cell Signaling Technology, cat#9661S), CTLA-4 (Abcam, cat# ab210254, Cambridge, UK), PD-1 (Abcam, cat# ab52587), PD-L1 (Cell Signaling Technology, cat# 13684S), RIP3 (Cell Signaling Technology, cat# 13526S), phospho-RIP3 (Cell Signaling Technology, cat# 93654S), MLKL (Cell Signaling Technology, cat# 14993S), phospho-MLKL (Cell Signaling Technology, cat# 91689S), STAT1 (Cell Signaling Technology, cat# 9172S), phospho-STAT1 (Y701) (Cell Signaling Technology, cat# 7649S), phospho-STAT1 (Y727) (Cell Signaling Technology, cat# 8826S). Image J software (NIH, Bethesda, MD, USA) was used for densitometry of Western blotting.

### 4.4. Knockdown of PD-L1 by siRNA

The detailed procedures were previously described [52]. Briefly, one day prior to siRNA transfection, 1 × 10^5^ THLE-2 cells were seeded on 6-well plates. In the next day, 25 nM siRNA was transfected using Lipofectamine 3000 (Thermo Fisher Scientific, cat# L3000-015), and 48 h after transfection, the cells were subjected to treatment of atezolizumab. After 24 h treatment, the WCLs were subjected to a Western blotting analysis. The following siRNAs, purchased from Dharmacon in Horizon Discovery (Lafayette, CO, USA), were used in the manuscript: human PD-L1 (cat# L-015836-01-0005, siRNAs: GCCGACUACAAGCGAAUUA, GGCAUUUGCUGAACGCAUU, GAAAAUGGAACCUGGCGAA, CCUAUAUGUGGUAGAGUAU), and nontargeting control (cat# D-001819-10-20, siRNAs: UGGUUUACAUGUCGACUAA, UGGUUUACAUGUUGUGUGA, UGGUUUACAUGUUUUCUGA, UGGUUUACAUGUUUUCCUA).

### 4.5. Cell Growth, LDH, ALT, and MTT Assays

For cell growth assay, the detailed procedures were previously described [53]. Briefly, 5 × 10^4^ THLE-2 or THLE-3 cells were seeded on 12-well plates (Corning) and incubated in the cell culture media containing 10% FBS for three days. Then, the cell culture media were changed to fresh media containing 0, 200, or 400 ug/mL atezolizumab and cells were cultured for additional 48 h. Subsequently, cell number was counted using a TC20 automated cell counter (Bio-Rad, Hercules, CA, USA).

For LDH assay, 1 × 10^4^ cells, 0.5 × 10^5^ cells, and 5 × 10^5^ THLE-2 cells were seeded on 96-well, 12-well, and 6-well plates, respectively, or 2 × 10^5^ human primary hepatocytes (HPH) cells were seeded on 48-well plates. Subsequently, they were cultured overnight. The next day the cells were treated with indicated concentration of atezolizumab as described in the manuscript. Five, twenty-four, and forty-eight hours after the drug treatment, the condition medium was subjected to lactate dehydrogenase (LDH) assay. The LDH assay was performed using LDH-Glo cytotoxicity assay (Promega, cat# J2381, Madison, WI, USA) according to the manufacturer’s instruction. Luminescence was detected using plate readers (Promega, GloMax discover and PerkinElmer, EnSight Multimode Plate Reader).

For cell viability, MTT (3-(4,5-dimethlthiazol-2-yl)-2,5-diphenyltetrazolium bromide) assay (Fisher Thermo Scientific, cat# M6494, Waltham, MA, USA) was performed according to manufacturer’s instructions. Briefly, 2 × 10^5^ THLE-2 cells were seeded on 96-well plates one day prior to cell treatment, the next day the cells were treated with different doses of atezolizumab as described in the manuscript.

ALT activity assays were developed based on quantification of pyruvate which is converted from alanine and α-ketoglutarate by ALT activity [54,55]. Detailed method will be provided upon request. Briefly, 4 × 10^4^ MDA-MB-231 cells were seeded on 96-well plates and cultured overnight. Next day, the cells were treated with 0.5 µM doxorubicin, 200 µg/mL ATE, or 200 µg/mL IPIL for 5 and 24 h. ALT release assay consists of measuring released ALT and total ALT. For measuring released LDH (Rr 40 µL), 40 µL of test sample was added to a well of 96-well plates, and 200 µL of reaction buffer containing L-alanine (Sigma-Aldrich, cat# A7627), PPA (Sigma-Aldrich, cat# 82870), NADH (Sigma-Aldrich, cat# 43420), LDH (Sigma-Aldrich, cat# L1254), and α-ketoglutarate (Sigma-Aldrich, cat# 75892) was added to each test sample, and the wells were measured by A340 at time point 15 min. For measuring total LDH (Rt 63 µL), 3 µL of 20% Triton X-100 was added to the same wells of the 96-well plates, and the 96-well plates were incubated at 37 °C for 1 h, and then the same wells were again measured by A340 at time point 1 h. Released ALT was calculated using the following formula:

Released ALT = Rate for released LDH (Rr 100 µL)/(Rate for released LDH (Rr 40 µL) + Rate for total LDH after Triton X-100 treatment (Rt 63 µL)).

### 4.6. Flow Cytometry

The detailed procedures were previously described [56]. THLE-2 cells (2 × 10^5^) were treated with 100 µg/mL IPIL or 3:1 ratio of THLE-2 cell culture medium:T-CM. Twenty-four hours after the treatment, THLE-2 cells were trypsinized, pelleted, and washed 2× with 1× FACS buffer (1 × PBS + 1% FBS). The THLE-2 cells were then stained with the following antibodies including IgG isotype controls: PD-L1 FITC (BD Bioscience, cat# BD558065, San Jose, CA, USA), mouse IgG1k FITC (BD Bioscience, cat# 555748), CTLA-4 PE (Abcam, cat# ab210254), and mouse IgG2a PE (Abcam, cat# ab91363). Samples were analyzed with BD LSR Flow cytometer using 488, 575 filters. The data were analyzed using FlowJo software (FlowJo LLC, Ashland, OR, USA). Flow cytometry data shown in the figures of this manuscript are representative of at least two independent experiments. Each cell treatment was coming from a single well.

### 4.7. Immunofluorescence

The detailed procedures were previously described [52]. THLE-2 cells (0.5 × 10^5^) were plated on fibronectin (10 µg/mL at 4 °C overnight, Sigma-Aldrich, cat# F1141-5MG) pre-coated glass cover slips on a 12-well plate and cultured overnight. Cells were then fixed in 4% paraformaldehyde (Electron Microscopy Science, cat# 15710, Hatfield, PA, USA) for 20 min and permeabilized with 0.2% Triton X-100/TBS or 0.5% Saponin (EMD Millipore)/TBS for 10 min. After blocking with 10% donkey serum (Jackson ImmunoResearch, cat# 017-000-121, Chester County, PA, USA) at room temperature for 1–2 h, cells were subjected to fluorescent immunostaining. ProLong Gold antifade reagent with DAPI (Thermo Fisher Scientific, cat# P36935) was used for mounting specimens on glass slides and nuclear staining. Images were captured by an LSM880 confocal microscope (Carl Zeiss Microscopy, LLC, White Plains, NY, USA).

### 4.8. Statistical Analysis

GraphPad Prism version 9.4.0. was used for statistical studies. Statistical significance was determined by two-way ANOVA followed by Tukey’s test and Student’s *t*-test (* *p*-value < 0.05; ** *p*-value < 0.01; *** *p*-value < 0.001; **** *p*-value < 0.0001). Data are expressed as mean ± SEM.

## 5. Conclusions

In summary, we demonstrated that the immune checkpoint inhibitor atezolizumab induced necroptosis and contributed to the hepatotoxicity of PD-L1^+^-human hepatocytes. The conditioned medium derived from the activated T cells also contributed to the hepatotoxicity via the necroptosis. The necrosome inhibitor necrostain-1 blocked both the atezolizumab and the hepatotoxicity induced by the conditioned medium derived from the T cells-. Taken together, our discovery provides a novel therapeutic strategy to reduce the hepatotoxicity induced by the immune checkpoint inhibitor, using a combination therapy of immune checkpoint inhibitors combined with necrostain-1.

## Data Availability

Research data will be provided upon request.

## Supplementary Materials

The following supplementary materials are available online at https://www.mdpi.com/article/10.3390/ijms241411694/s1.
